# Antidepressant Effect of Crocin in Mice with Chronic Mild Stress

**DOI:** 10.3390/molecules27175462

**Published:** 2022-08-25

**Authors:** Walaa F. Alsanie, Abdulhakeem S. Alamri, Osama Abdulaziz, Magdi M. Salih, Abdulwahab Alamri, Syed Mohammed Basheeruddin Asdaq, Mohammed Hisham Alhomrani, Majid Alhomrani

**Affiliations:** 1 Department of Clinical Laboratory Sciences, The Faculty of Applied Medical Sciences, Taif University, Taif 21944, Saudi Arabia; 2 Centre of Biomedical Sciences Research (CBSR), Deanship of Scientific Research, Taif University, Taif 21944, Saudi Arabia; 3 Department of Pharmacology and Toxicology, College of Pharmacy, University of Hail, Hail 81451, Saudi Arabia; 4 Department of Pharmacy Practice, College of Pharmacy, AlMaarefa University, Dariyah 13713, Saudi Arabia; 5 College of Medicine, King Abdulaziz University, Jeddah 21589, Saudi Arabia

**Keywords:** antidepressant, antioxidants, chronic mild stress, crocin, corticosterone, nitrite, tail suspension test

## Abstract

This study aimed to investigate the antidepressant property of crocin (Crocetin digentiobiose ester) using a chronic mild stress (CMS)-induced depression model in experimental mice. The tail suspension test (TST) and the sucrose preference test were used to evaluate the antidepressant effect on albino mice of either sex after three weeks of CMS. The period of immobility in the TST and percentage preference for sucrose solution were recorded. By monitoring brain malondialdehyde (MDA) level, catalase (CAT) activity, and reduced glutathione (GSH) level, the antioxidant potential was assessed. Three dosages of crocin (4.84, 9.69, and 19.38 mg/kg) were evaluated. When compared to controls, animals that received crocin administration during three periods of CMS had considerably shorter immobility times during the TST. Crocin treatment also raised the percentage preference for sucrose solution in a dose-dependent manner, bringing it to parity with the conventional antidepressant, imipramine. Animals that received a high dose of crocin had a much greater spontaneous locomotor activity. Furthermore, a high dose of crocin remarkably lowered plasma corticosterone and nitrite levels brought on by CMS. Additionally, high doses of crocin given during CMS greatly enhanced reduced glutathione levels while considerably reducing the brain’s MDA and catalase activities. In conclusion, high doses of crocin may have an antidepressant effect in an animal model through several mechanisms. However, further studies should be carried out to explore the role of neurotransmitters for their antidepressant property.

## 1. Introduction

Depression is a psychiatric disease marked by poor mood, loss of interest in regular activities, anhedonia, feelings of worthlessness, sleep difficulty, and suicidal impulses [1]. Monoamine neurotransmitter abnormalities [2], as well as enhanced oxidative and nitrosative damage [3], are the main mechanisms. The monoamine hypothesis states that depression is caused by a depletion of monoamines such as serotonin, norepinephrine, and dopamine in the hippocampus, limbic system, and frontal cortex [4]. The major enzyme involved in the metabolism of these monoamines is monoamine oxidase (MAO). Patients with significant depression had lower antioxidant levels, as well as increased oxidative and nitrosative stress [5].

Corticotrophin-releasing hormone hypersecretion and glucocorticoid response are impaired in depression [6]. Around 50% of depressive people (80% if seriously depressed) have hyperactivity in the hypothalamic–pituitary–adrenal axis. The hypothalamic–pituitary–adrenal axis hyperactivity changes when animals are exposed to chronic stress [7]. Stress plays a significant influence in the development of depression in humans [8]. Depressive symptoms arising in laboratory animals exposed to chronic mild stress (CMS) are similar to human depression. Anhedonia, food intake disorder, cognitive deficiencies, decreased sexual activity, longer immobility times during the forced swim test (FST) and tail suspension test (TST), and heightened anxiety are just a few of the behavioural abnormalities that animals exhibit when depressed [9]. Increased brain oxidative stress caused by CMS was thought to be a primary factor in neurotoxicity and neuronal death, which may contribute to the development of chronic stress-induced depression [10,11]. Recent studies found that oxidative stress occurred in animals under adverse conditions [12,13,14].

Antidepressants are thought to work on the central monoaminergic systems, primarily serotonergic and nor-adrenergic synaptic neurotransmission, in major depression. The most typically given medicines are selective serotonin-reuptake inhibitors such as paroxetine and fluoxetine, as well as specialized serotonin-noradrenaline reuptake inhibitors such as reboxetine and desipramine [15]. Even though they are successful in treating most depressive episodes, a considerable proportion of depressed individuals do not show symptoms of improvement until 2–3 weeks after starting treatment. In addition, around a third of these individuals respond to treatment either partially or not at all [16]. Moreover, these medications might cause sedation, anticholinergic effects, seizures, impotence, postural hypotension, anxiety, dizziness, respiratory issues, weight gain, cheese response, cardiac dysrhythmias, insomnia, agitation, and fatigue [17]. As a result, one option is to explore natural products from plant sources and their bioactive elements for antidepressant efficacy.

*Crocus sativus* L., often known as saffron, is a perennial stemless herb in the Iridaceae family that is mostly farmed in Spain and Iran, but also in Greece, Turkey, Azerbaijan, France, Italy, India, and China on a lesser level [18]. In folk medicine, saffron was used as an antispasmodic, eupeptic, gingival sedative, anticatarrhal, nerve sedative, carminative, diaphoretic, expectorant, stimulant, stomachic, and aphrodisiac [19]. Saffron extracts and active components have anticonvulsant [20], antidepressant [21], anti-inflammatory [22], and antitumor properties [23]. Saffron’s potential pharmacological effects are attributable to the presence of safranal (aromatic terpene), carotenoids crocetin (mono and diglycosyl esters of polyene dicarboxylic acid) and crocins (digentiobiosyl ester of crocetin) [24]. Crocin exhibits hypolipidemic [25,26], anticancer [27,28], antiulcer [29], and antioxidant [30,31] properties. It has also been suggested that it has cardioprotective potential [32]. In acute preclinical trials, the antidepressant effects of various extracts of C. sativus L. stigmas, tepals, and corms, as well as their active ingredients, were investigated and found to be considerably more helpful than placebo [33,34,35,36]. Our purpose in this work was to evaluate the impact of crocin in alleviating depression-like behaviour in animal experimental models and to correlate this with their antioxidant potential.

## 2. Results

### 2.1. The Effect of Crocin and Imipramine on Period of Immobility

**Figure 1:**
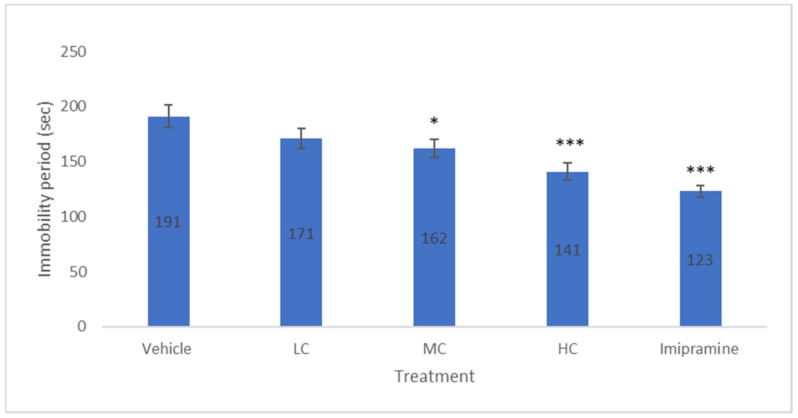
The changes in immobility period (sec) due to crocin and imipramine. Data were given as mean and standard error of mean. Each group had eight animals. Statistical analysis was performed by one-way analysis of variance (ANOVA) and post-ANOVA Tukey’s test; * *p* < 0.05, and *** *p* < 0.001 when compared to vehicle; LC—low dose of crocin; MC—medium dose of crocin; HC—high dose of crocin.

The animals of all five groups were given chronic mild stress to develop depression. A low dose of crocin caused a slight decrease in the period of immobility but it was not significant. Medium (*p* < 0.05) and high (*p* < 0.001) doses of crocin, dose-dependently produced a significant depletion in the period of immobility (Figure 1). Further, a standard tricyclic antidepressant (imipramine) also exhibited a significant (*p* < 0.001) reduction in the immobility period on par with the high dose of crocin.

### 2.2. The Effect of Crocin and Imipramine on Sucrose Preference Model

**Figure 2:**
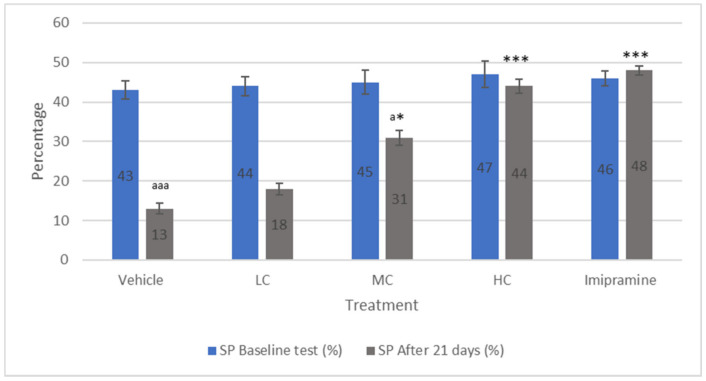
The changes in percentage sucrose preference test due to crocin and imipramine. Data were given as mean and standard error of mean. Each group had eight animals. Statistical analysis was performed by one-way analysis of variance (ANOVA) and post-ANOVA Tukey’s test; * *p* < 0.05, and *** *p* < 0.001 when compared to vehicle; ^a^ *p* < 0.05, and ^aaa^ *p* < 0.001 when compared to baseline percentage sucrose preference results of the same group; LC—low dose of crocin; MC—medium dose of crocin; HC—high dose of crocin.

The preference of animals to sucrose was recorded at the beginning of the treatment period as well as at the end of 21 days of chronic mild stress (CMS). No significant alteration was recorded among different groups after the first reading (Figure 2), while there was a significant (*p* < 0.001) decline in the preference of sucrose at the end of 21 days of CMS in vehicle-treated groups compared to their own baseline values. Further, a low dose of crocin failed to prevent a significant (*p* < 0.001) decline in the preference of sucrose compared to their first reading. Treatment of animals with a medium dose of crocin resulted in a significant (*p* < 0.05) increase in sucrose preference compared to the vehicle-treated group. However, it continued to be significantly (*p* < 0.05) low when compared to their baseline preference for sucrose. Both a high dose of crocin and imipramine produced a significantly (*p* < 0.001) increased percentage in sucrose preference compared to the vehicle-treated group. Further, no significant difference in sucrose preference was observed in these groups when compared to their initial reading prior to the onset of CMS.

### 2.3. The Effect of Crocin and Imipramine on Locomomotor Activity

**Figure 3:**
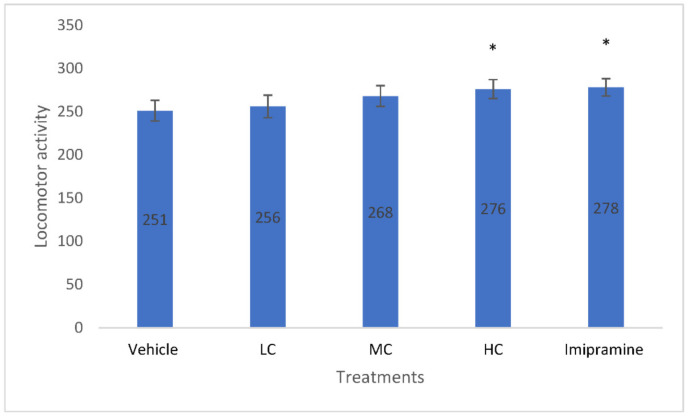
The changes in number of locomotor activity due to crocin and imipramine. Data were given as mean and standard error of mean. Each group had eight animals. Statistical analysis was performed by one-way analysis of variance (ANOVA) and post-ANOVA Tukey’s test; * *p* < 0.05 when compared to vehicle; LC—low dose of crocin; MC—medium dose of crocin; HC—high dose of crocin.

A significantly (*p* < 0.05) increased locomotor activity was noticed in animals that received a high dose of crocin and imipramine, compared to the vehicle-treated group (Figure 3). On the contrary, no significant change in locomotor activity was noticed in animals that received low or medium doses of crocin.

### 2.4. The Effect of Crocin and Imipramine on Plasma Nitrite and Corticosterone

**Figure 4:**
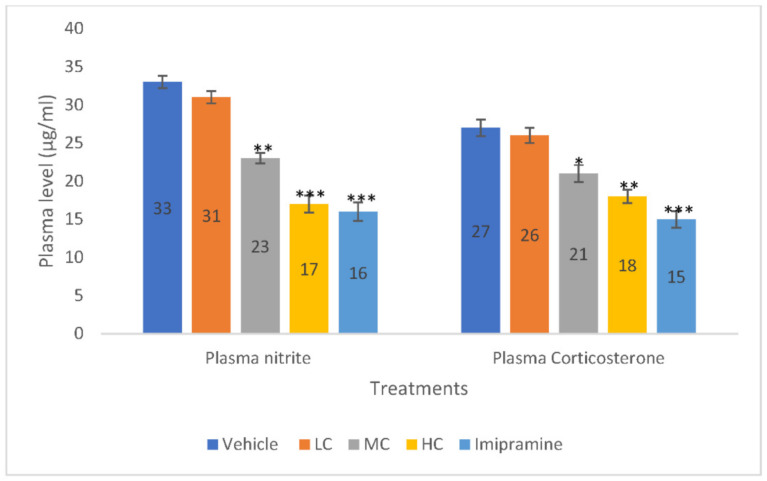
The changes on plasma nitrite and corticosterone levels due to crocin and imipramine. Data were given as mean and standard error of mean. Each group had eight animals. Statistical analysis was performed by one-way analysis of variance (ANOVA) and post-ANOVA Tukey’s test; * *p* < 0.05, ** *p* < 0.01, and *** *p* < 0.001 when compared to vehicle; LC—low dose of crocin; MC—medium dose of crocin; HC—high dose of crocin.

Both medium (*p* < 0.01) and high (*p* < 0.001) doses of crocin, dose-dependently produced significant reduction in plasma nitrite level compared to the vehicle-treated group. The administration of imipramine also caused a significant (*p* < 0.001) decrease in plasma nitrite level, like a high dose of crocin (Figure 4). However, no significant change in plasma nitrite level was noticed with a low dose of crocin when compared to the vehicle control group.

Further, plasma corticosterone level was significantly (*p* < 0.01) declined in animals that received a high dose of crocin. However, the standard tricyclic antidepressant, imipramine, produced a stronger significant (*p* < 0.001) reduction in plasma corticosterone than a high dose of crocin. Further, a medium dose of crocin also showed a modest, but significant (*p* < 0.05) decline in the plasma corticosterone level when compared to the vehicle-treated group (Figure 4).

### 2.5. The Effect of Crocin and Imipramine on Brain Malondialdehyde (MDA) Level

**Figure 5:**
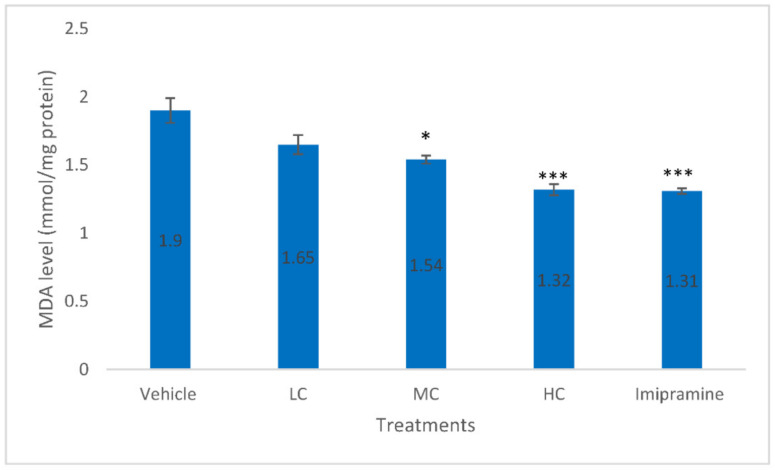
The changes on brain MDA level due to crocin and imipramine. Data were given as mean and standard error of mean. Each group had eight animals. Statistical analysis was performed by one-way analysis of variance (ANOVA) and post-ANOVA Tukey’s test; * *p* < 0.05, and *** *p* < 0.001 when compared to vehicle; LC—low dose of crocin; MC—medium dose of crocin; HC—high dose of crocin.

As shown by Figure 5, brain MDA level was significantly reduced in animals that received a medium dose of crocin (*p* < 0.05), high dose of crocin (*p* < 0.001) and imipramine (*p* < 0.001) when compared to the vehicle-treated group at the end of CMS. The effect was found to be modest with a medium dose of crocin whereas, both a high dose of crocin and imipramine showed almost similar reduction in brain MDA level.

### 2.6. The Effect of Crocin and Imipramine on Brain Catalase Activity

**Figure 6:**
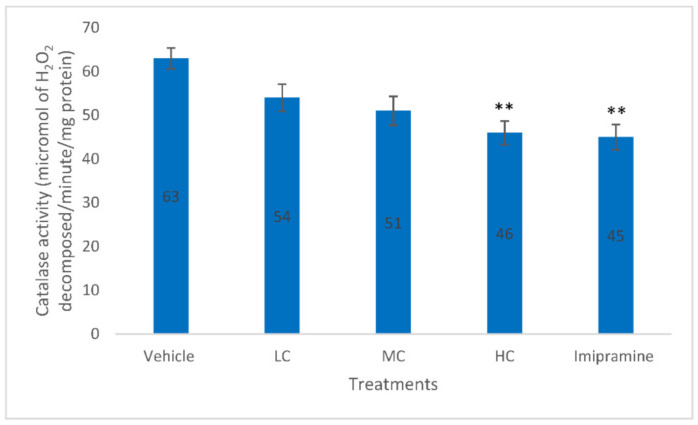
The changes on brain catalase activity due to crocin and imipramine. Data were given as mean and standard error of mean. Each group had eight animals. Statistical analysis was performed by one-way analysis of variance (ANOVA) and post-ANOVA Tukey’s test; ** *p* < 0.01 when compared to vehicle; LC—low dose of crocin; MC—medium dose of crocin; HC—high dose of crocin.

As described by Figure 6, only a high dose of crocin and imipramine were able to significantly (*p* < 0.01) reduce the brain catalase activity when compared to the vehicle-treated group. Although there was reduction in catalase activity with low and medium doses of crocin, this was not significant.

### 2.7. The Effect of Crocin and Imipramine on Brain Glutathione (GSH) Level

**Figure 7:**
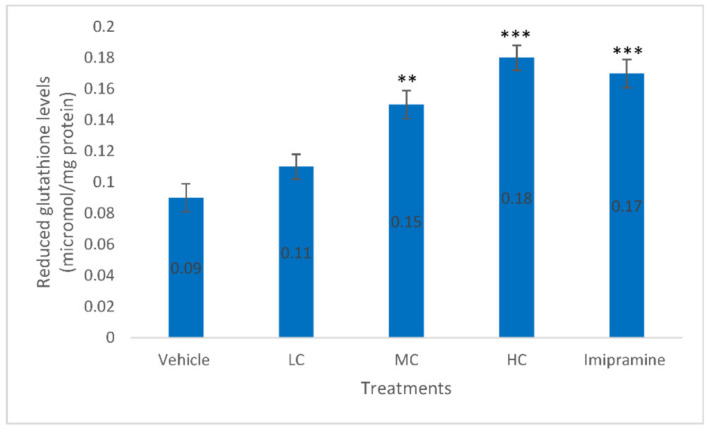
The changes on brain reduced glutathione levels due to crocin and imipramine. Data were given as mean and standard error of mean. Each group had eight animals. Statistical analysis was performed by one-way analysis of variance (ANOVA) and post-ANOVA Tukey’s test; ** *p* < 0.01, and *** *p* < 0.001 when compared to vehicle; LC—low dose of crocin; MC—medium dose of crocin; HC—high dose of crocin.

Administration of animals with a medium dose of crocin (*p* < 0.01), high dose of crocin (*p* < 0.001) and standard antidepressant, imipramine (*p* < 0.001) produced significantly elevated brain SGH levels compared to vehicle-treated animals (Figure 7). The best increase in brain SGH level was found with a high dose of crocin, rather than standard imipramine.

## 3. Discussion

The development of more efficient and effective antidepressants may be aided by the quest for new antidepressants based on novel methodologies. Recent studies have placed a greater emphasis on natural goods as they looked for safe and effective antidepressant medications [37,38]. The purpose of this study was to investigate the impact of crocin in relieving depression produced by prolonged mild stress in an animal experimental system. The study’s findings demonstrated that crocin had dose-dependent antidepressant properties, perhaps as a result of its antioxidant capability. Crocin’s antidepressant effects were comparable to those of imipramine, a common tricyclic antidepressant. Chronic mild stress (CMS) is a widely used paradigm to cause depressive behaviour in mice that is like the depressive pattern seen in people who are exposed to various stressors during a normal day [11]. Two behavioural models that are regularly employed to evaluate prospective antidepressants are the tail suspension test (TST) and the sucrose preference test [39]. In our TST set-up, chronic mild stress led to a noticeably longer period of immobility, which was reduced by both high doses of crocin and imipramine. Additionally, when compared to control, imipramine and high doses of crocin remarkably increased the locomotor activity of CMS mice, indicating their CNS stimulant action.

Another technique utilised to evaluate the antidepressant potential of crocin in CMS mice was the sucrose preference test. This test is designed to identify anhedonia-like behaviour, which is characterised by a subject’s loss of interest in or satisfaction in ordinarily pleasurable or happy activities. It is one of the most prevalent signs of depression in people [11]. We found that mice demonstrated a high preference for sucrose before the onset of CMS. The percentage of sucrose preference had, however, diminished significantly three weeks following CMS. A dose-dependent restoration of sucrose preference percentage occurred after crocin administration during CMS. When compared to the control, the recovery brought about by a high dose of crocin was comparable to that brought about by imipramine, while low and moderate doses of crocin likewise considerably improved sucrose preference. This finding implies that crocin had a dose-dependent, antidepressant-like activity.

Plasma glucocorticoid levels will change when the hypothalamic–pituitary–adrenal (HPA) axis is active, which may cause depression [40]. According to Sousa et al. [41], elevated cortisol level may influence the development of depressive symptoms by affecting various mental processes. Chronic antidepressant use is known to lower HPA activity, which returns the HPA axis to its natural state [41]. According to findings from another study, mild chronic stress increases plasma corticosterone levels via hyperactivating the HPA axis [42]. In our experiment, high doses of crocin and imipramine treatment both reduced the hyperactivity of the HPA axis brought on by CMS in mice, as seen by a significant decrease in plasma corticosterone levels in stressed mice.

Additionally, the stress brought on by CMS causes the body to produce oxygen free radicals, which are shown as a rise in blood nitrite levels [43]. High doses of crocin given to mice during CMS resulted in a considerable drop in plasma nitrite levels, indicating a decrease in nitrosative stress. Thus, crocin significantly defended the animals from oxidative damage brought on by CMS, which is commonly seen with many natural substances [44].

Free radicals from oxygen contribute to serious depression. Reactive oxygen species formation, lipid peroxidation, and reduced antioxidant enzyme activities may be caused by immune-inflammatory process activation, anomalies in lipids, and these processes may be linked to depression [43]. It has been shown that 4-tert-butylphenol-induced stress increased MDA level, decreased GSH activity, and caused a decline in antioxidant capability [45]. CMS depletes the brain’s antioxidant capacity, perhaps via producing reactive oxygen species [46]. In the brain, we found that CMS increased lipid peroxidation and catalase activity while decreasing reduced glutathione levels. These characteristics were markedly reversed after receiving crocin for three consecutive weeks. Considering this, crocin demonstrated strong antioxidant action in mice. In our experiment, we found that plasma nitrite levels were markedly elevated in CMS-exposed mice [46]. By lowering the plasma nitrite levels in stressed mice, crocin greatly reduced nitrosative stress. Therefore, crocin greatly shielded the mice from the oxidative stress brought on by CMS. Therefore, we speculated that crocin had the potential to be antidepressant in animal models at high dosages via a variety of mechanisms, most likely through boosting antioxidant potential, re-establishing the HPA axis, and removing free radicals.

As previously stated, the pharmacological properties of saffron are attributed to safranal, crocins, and crocetin. The molecule of this study, crocin, was reported to be devoid of any marked damage to the major organs of the body when tested in mice [47] and established to be safe for human consumption [48]. The crocin family consists of a crocetin moiety linked to sugars via an ester bond [49]. Currently, mass spectrometric data identified 16 crocins in both cis- and trans-forms [50]. These 16 isomers are mono- or di-glycosylated crocetin, whereas trans-4-GG crocetin is the main crocin in saffron [51]. Furthermore, trans-4-GG crocin is a water-soluble carotenoid that is mostly hydrolysed by intestinal enzymes and microbiota into deglycosylated trans-crocetin, which is passively absorbed by the mucosal layer of the intestine [52,53]. This trans-crocetin isomer is primarily responsible for crocin’s pharmacological activity when taken orally [54]. In terms of pharmacological effects, however, a study found that administering crocin intraperitoneally was superior to administering it orally, most likely due to bypassing first-pass metabolism and gastric hydrolysis, resulting in improved bioavailability and enhanced permeation through the blood brain barrier and localization of crocin inside the brain [55,56]. Therefore, further investigation is required to examine the differences in crocin’s antidepressant activity when administered orally versus intraperitoneally.

## 4. Materials and Methods

### 4.1. Experimental Animals

In accordance with accepted animal care practices, albino mice (both sexes, 20–25 g, 10–14 weeks old) were housed in an air-conditioned room (23–25 °C) with alternating light and dark cycle of 12 h each. To prevent affecting the animals’ behavioural patterns, all variables were maintained throughout the experiment. A standard rodent diet provided by a local wholesaler was used to feed them. During the light phase (9 am to 10 am) of the light and dark daily cycle, the animals received their respective treatments. Prior to starting the study, the local ethics committee approved (211/022/IRB) the study proposal.

### 4.2. Materials

Imipramine hydrochloride was supplied by Sigma-Aldrich. Crocin (Crocetin digentiobiose ester, product number 17304) was obtained from Sigma Aldrich Co (Darmstadt, Germany). All chemicals and study kits were acquired from standard sources. To dissolve imipramine hydrochloride, ordinary saline was utilised. Based on the concentration of crocin found in 25, 50, and 100 mg of saffron, three doses of crocin (4.84, 9.69, and 19.38 mg/kg) were chosen. This was achieved utilizing a dose response study using high performance liquid chromatography, as described in our prior work [57].

### 4.3. Experimental Grouping

All the mice utilized in the study were split into ten groups with eight mice in each group. Carboxy methyl cellulose (CMC) was given to group I as a vehicle, while groups II, III, and IV received doses of crocin of 4.84 mg/kg (low dosage of crocin—LC), 9.69 mg/kg (medium dose of crocin—MC), and 19.38 mg/kg (high dose of crocin—HC), respectively. The group V animals received imipramine (15 mg/kg). For three weeks, all treatments were administered orally 30 min prior to the onset of stress (chronic mild stress—CMS) (21 days). On the 21st day, the mice’s locomotor activity was assessed after being exposed to stress for 60 min. On the 22nd day, a TST was performed on the mice.

Animals in groups VII, VIII, and IX received the treatments LC, MC, and HC, respectively, while those in group VI received the vehicle therapy (CMC). Animals in group X received imipramine (15 mg/kg). Every therapy was administered orally for three weeks prior to CMS. On the 21st day, a sucrose preference test was conducted 60 min after CMS.

### 4.4. Chronic Mild Stress (CMS)

Depression may result from neuronal toxicity brought on by persistent stress. According to a previously documented approach, the mice were subjected to mild chronic stress [58,59]. Mice were subjected to each type of stress pattern once per day for three weeks. Thirty minutes prior to the introduction of the stress pattern, the medications were administered to the appropriate groups. Chronic stress can cause neuronal toxicity, which can lead to depression. The mice were given chronic mild stress in accordance with a previously described procedure [59]. The different stress patterns were induced in mice once a day for three weeks. The drugs were given to the respective groups 30 min before the induction of the stress pattern.

In the first week of CMS, animals were immobilised for 2 h on day 1, introduced to empty water bottles on day 2 for 1 h, subjected to foreign bodies for 24 h on day 3, kept under nocturnal illumination on day 4, tilted cage at 45° for 7 h on day 6, and tail pinch (30 s) on day 7. The stress patterns’ order was altered throughout weeks two and three.

### 4.5. Tail Suspension Test (TST)

The established TST was conducted according to a methodology that has been thoroughly reported elsewhere [58], and immobility time was recorded. Using adhesive tape in a dimly lit environment, mice were briefly hung by their tails 60 cm above the floor. For the latter four minutes of a six-minute session, immobility was noted. When mice hung passively and motionlessly, they were perceived as immobile. Both control and test group animals were subjected to this test to determine the influence of intervention on immobility time.

### 4.6. Measurement of Locomotor Activity

Using a photoactometer, the horizontal locomotor activity ratings of control and test animals were recorded for 5 min [58]. Each mouse was maintained in the device for five minutes. If the mouse engaged in any exploratory behaviour, the light’s beam would interrupt, and the instrument would automatically record the activity’s duration on its digital recorder. Digital recordings ceased recording as soon as the animal paused its activities.

### 4.7. Sucrose Preference Test

Animals were trained to consume sucrose solution while fasted for two days prior to exposing them to persistent mild stress. Three days later, after a 23-h fast, the animals were introduced to two bottles, one containing regular water and the other containing sucrose solution. The baseline percentage of sucrose solution preference was computed [58]. The test was repeated after 21 days of therapy to ascertain the impact of therapy on the subjects’ preference for sucrose solution as a percentage, which will serve as an indicator for depression brought on by stress.

### 4.8. Biochemical Estimations in Plasma

Blood was collected on day 23 and centrifuged to separate plasma for nitrite and corticosterone measurement [60,61]. This was performed 60 min after the treatment was provided [48].

### 4.9. Biochemical Estimations in Brain Homogenate

On the 23rd day, the mice were decapitated, and their brains were isolated after blood samples were taken. The obtained brain samples were washed with cold buffer (pH 7.4) consisting of 0.25 M sucrose, 0.1 M Tris, and 0.02 M ethylenediamine tetraacetic acid. The brain samples were centrifuged. The concentrations of catalase, reduced glutathione, and lipid peroxidation were measured in the centrifuged supernatant. Using a semi-automatic autoanalyzer, the total protein kit (Siemens, Siemens Ltd., Mumbai, India) was used to measure the amount of total protein in brain homogenate (Chem plus-V2, Mannheim, Germany), while MDA level, reduced glutathione, and catalase activity were determined following the procedures reported by Wills [62], Jollow et al. [63], and Claiborne [64], respectively, using UV–visible spectrophotometers.

### 4.10. Statistical Evaluation

Each group contained eight animals, which were utilised to gather the data for the analysis. A one-way analysis of variance (ANOVA) and the Tukey test were used to assess the data (Graphpad Prism, San Diego, CA, USA). The data in the tables were expressed as mean ± SEM, and differences were deemed significant when the *p*-value difference between groups was less than 0.05.

## 5. Conclusions

According to the results of the present investigation, crocin may have antidepressant characteristics at high doses that are comparable to those of the widely used antidepressant, imipramine. Crocin had antioxidant and corticosterone-restorative properties, and it may also have an antidepressant effect due to an increase in brain glutathione level. However, additional study is required to determine how neurotransmitters, among other pathways, contribute to the antidepressant effect of crocin.

## Data Availability

Not applicable.
